# Effects of date fruit (*Phoenix dactylifera L.*) on labor and delivery outcomes: a systematic review and meta-analysis

**DOI:** 10.1186/s12884-020-02915-x

**Published:** 2020-04-14

**Authors:** Alireza Bagherzadeh Karimi, Asghar Elmi, Mojgan Mirghafourvand, Roghaiyeh Baghervand Navid

**Affiliations:** 1 Department of Persian Medicine, School of Traditional Medicine, Tabriz University of Medical Sciences, Tabriz, Iran; 2 School of Medicine, Tabriz University of Medical Sciences, Tabriz, Iran; 3 Midwifery Department, Social Determinants of Health Research Center, Tabriz University of Medical Sciences, Tabriz, Iran

**Keywords:** Date palm, Date fruit, Phoenix dactiylifera, Delivery, Labor, Systematic review, Meta-analysis

## Abstract

**Background:**

The rate of cesarean section is increasing in all over the world with different drafts in various countries. This growth increases unpleasant outcomes of delivery. Recent studies explained the benefits of date palm fruit on labor process improvement. Date fruit can be considered as a factor for increasing vaginal delivery and also reducing the frequency of caesarean section in order to prevent its great complications. This systematic review has been designed to review clinical studies that investigate the effects of date palm fruit on labor outcomes (duration of labor stages, bishop score, and frequency of cesarean section) compared with routine cares.

**Methods:**

This study was performed in 2019. Required data has been collected from electronic databases and manual searches. All randomized clinical trials evaluating the effects of date palm fruit on labor and delivery that were published from January 2000 to August 2019 in English and Persian languages, were incorporated in this systematic review. The methodological quality of the included studies was evaluated according to the risk of bias assessment of Cochrane handbook of systematic reviews, and were then reported using Preferred Reporting Items for Systematic Reviews and Meta-Analysis (PRISMA) statement.

**Results:**

Eight studies were included in the qualitative and quantitative synthesis. Meta-Analysis showed that date fruit consumption can significantly reduce active phase of labor (three trials with 380 participants; (MD = − 109.3, 95%CI (− 196.32, − 22.29; I^2^ = 89%), *P* = 0.01), and also it can significantly improve the bishop score (two trials with 320 participants; MD = 2.45, 95%CI (1.87, 3.04; I^2^ = 0%), *P* < 0.00001). Date fruit consumption had no effects on the duration of first, second, and third stages of labor, and the frequency of cesarean section.

**Conclusion:**

Date can reduce the duration of active phase and improve the bishop score; however, due to from the low to mediate quality of the studies; it seems that the other studies are needed to prove these results better than this.

## Background

### Delivery and its process

Since 1970 A. D, rate of caesarean section has increased in countries with high income. However, this rate in middle to low income countries increases with a specific rising draft [1, 2]. This increase is significant in developing countries such as Iran with the caesarean section rate of 48%. This prevalence rate is much more than global rate [3]. The growth of caesarean section rate increases unpleasant outcomes of delivery and neonatal complications such as respiratory distress syndrome, transient tachypnea of newborn and increased NICU admission [4, 5]. WHO (World Health Organization) has unnecessary announced the rate of caesarean section higher than 15% [4]. A few factors have led to the growth of caesarean section such as repeated caesarean, high maternal age in pregnancy, pregnancy with IVF (In vitro fertilization) or IUI (Intra uterine insemination), pregnancy after recurrent abortions, desire of physicians to caesarean section, and this belief that the prolapsed uterus and frequency do not occur after caesarean section [6–10]. Abnormal progress of labor and ineffective contractions of uterus are identified as two common reasons of caesarean section [11, 12].

According to the WHO, normal birth can be defined as:“ spontaneous in onset, low-risk at the start of labor, and remaining so throughout labor and delivery. The infant is spontaneously born in the vertex position between 37 and 42 completed weeks of pregnancy” [13] There will be different methods for labor induction if the progress of labor is not appropriate, which are used alone or in combination together similar to the strip of membranes and the usage of prostaglandins or oxytocin. These methods accelerate the labor progress and decrease the caesarean section frequency [14]. Nevertheless, a review study conducted on Cochrane explained that the pregnant women are not satisfied about their labor because of the difficulties of labor [15]. Most recently, the researchers have been paying attention to remove labor difficulties, and following that, to reduce the caesarean section frequency [16, 17]. Pregnant women need energy during labor; therefore the amount of required energy for active phase of labor should be 50–100 kcal (Kilo calories) [16, 17]. The drinking and eating are prohibited for pregnant women during labor in hospitals. It is important to know that it will lead to energy reducing, fatigue, and lack of cooperation in pregnant women, and finally could result in reducing the beneficial effects on mother and infant outcomes [12, 18, 19]. Scheepers et al. showed that the consumption of carbohydrate during labor can reduce the rate of augmented labor and decrease abnormal progress of labor [20].

### Date and its role in the labor

Among the studied sugars, is the date palm fruit; which is known as the scientific name of *Phoenix dactylifera* L. Date fruit contains different vitamins (riboflavin, biotin, thiamin, folic acid, and ascorbic acid), higher percentage of sugar and carbohydrates, proteins, fatty acids, salt and minerals such as potassium and magnesium [21, 22]. Date fruit due to energy production and having enough calories, can be helpful for pregnant women during labor, and it can also prevent physical weakness [23–25]. Furthermore, because it contains sugar, it is fast in digestion and absorption [25]. In addition to generating energy, date fruit also contains necessary and unnecessary fatty acids that can produce prostaglandins playing an important role in cervix ripening, acceleration of delivery progress, increase of uterine contractions, and inducing labor [26, 27]. In addition, date fruit contains any hormones that prepare uterine to stretching and child birth [28]. Also, date can accelerate labor process, increase cervix dilatation, and reduce the need for induction [23, 24]. Moreover, date in Traditional Persian Medicine (TPM) has been mentioned as a facilitator medicinal food for labor [29]. TPM is an ancient medical system and one of the important complementary and alternative medicines that has been utilized in Iran, India, and the middle east from ancient eras up to now [30, 31]. Nevertheless, there are few studies supporting the relationship between labor and date.

Most recently, a systematic review conducted on the effects of date fruit on pregnancy and delivery has been published [32]; however, there are some differences between its reviewed outcomes and this study. Therefore in that study the reporting bias and publication bias have not been regarded in inclusion of studies to meta-analysis. Accordingly this reason can cause wrong changes in meta-analysis results.

Due to the studies conducted on the benefits of date palm fruit on labor process improvement, date can be considered as a factor for increasing vaginal delivery and reducing the frequency of caesarean section in order to prevent its great complications. This systematic review aimed to review clinical studies that investigate the effects of date palm fruit on labor outcomes (duration of labor stages, bishop score, and frequency of cesarean section) compared to routine cares.

## Methods

This systematic review and meta-analysis was reported according to Preferred Reporting Items for Systematic Reviews and Meta-Analysis (PRISMA) statement. This systematic review was not registered on PROSPERO.

### Eligibility criteria

#### Inclusion and exclusion criteria of studies

All clinical trials including randomized and quasi-randomized, evaluating the effects of date palm fruit on labor and delivery that have been published from 2000 until August 2019 in English and Persian languages were included in this systematic review. Also, non-randomized clinical trials, editorials, reviews, books, case reports, case series, letter to editors, qualitative studies, and short communications were excluded. There was no limitation for the length of follow-up or treatment.

### Inclusion criteria (PICOS format)

1. Participants: all pregnant women in 36–42 weeks of pregnancy with no restrictions on patient’s age, nationality, and the number of parity without any serious complication.
2. Interventions: consumption of date (fruit or extract) without restriction in the number of the fruit and the duration of intervention.
3. Controls: The routine cares of pregnant women.
4. Outcomes: The primary outcomes were the duration of labor stages and the secondary outcomes were the frequency of caesarian section and bishop score.
5. Study type: Randomized and quasi-randomized clinical trials.

### Search strategy

This systematic review was performed in 2019 and the last search was conducted in August 2019. Data have been collected from the databases such as PubMed, Scopus, Web of Knowledge, Clinical Keys, Embase, Google Scholar Search Engine, Scientific Information Database (SID), and IRCT (Iranian Registry of Clinical Trials) to search the relevant articles published from January 2000 to August 2019 using the keywords *Phoenix dactylifera*, date palm, date fruit, labor, and delivery. Also, manual search of the reliable journal data-bases was accomplished, and the references in all review articles were checked for additional related articles. To search for unpublished articles (grey literature), European Association for Grey Literature Exploitation (EAGLE) and Health Care Management Information Consortium (HMIC) were investigated. The search strategy for PubMed is available in Appendix 1 (see Additional file 1). Investigated data was transferred to Endnote software.

### Study selection

The selected studies extracted from the databases by Endnote software, were independently evaluated by two authors (AE and RBN). Disagreements between them were referred to the third inspector (MM). At first, the titles of all papers were reviewed and inconsistent studies with the objectives of the study were excluded from the study. In the next step, abstract of chosen studies were reviewed and incompatible studies were excluded. Then, full texts of chosen studies were extracted. In the last stage, full-text articles were surveyed to exclude those that did not match with the inclusion criteria and the study aims. The authors elicited data from all eligible studies and registered the elicited data in the appropriate forms. Data for the primary objective of the review was gathered from the full text of each paper consisting of the trial name, year of publication, study design, sample size, participants, intervention protocol, used parts of plant, comparisons, results, and other characteristics.

### Assessment of risk of Bias

The methodological quality of the included studies and their risk of bias were independently evaluated by two reviewers (MM, ABK) using RevMan 5.3.0 software, in terms of the risk of bias assessment of the Cochrane handbook. The assessment criteria consisted of seven items as follows: Selection bias (allocation concealment); Selection bias (random sequence generation); Performance bias (blinding of participants and personnel); Detection bias (blinding of outcome assessors); Attrition bias (dropouts and exclusion addressing and intention to treat analysis); Reporting bias (selective or nonselective reporting); Other bias (registration of protocol, conflict of interest declaration, ethical criteria, inclusion and exclusion criteria, sample size calculating, and funding sources declaration). Each study was evaluated as High, Low, or Unclear risk of bias for each item. Any disagreements between the two reviewers were resolved by discussion with the corresponding author. Because the number of included studies in meta-analysis was lower than 10 studies, the graphical or statistical methods were not used to evaluate publication bias.

### Statistical analysis

The results of the studies were analyzed using “Review Manager” software (RevMan 5.3.0 provided by Cochrane Collaboration). The types of intervention in included studies were similar. We integrated the studies according to the types of outcomes (labor phases duration, frequency of caesarian section, and bishop score). We included all the intended outcomes of the different reports of each trial to the meta-analyses once. Dichotomous data were summarized as risk ratio (RR), and continuous data as mean difference (MD). Heterogeneity between the studies was evaluated using X^2^ (chi-squared) test and I^2^ statistic. I^2^ was used to assess heterogeneity between studies with ≥75%, 25–75, and < 25% which were considered as high, moderate, and low heterogeneity, respectively, to indicate a substantial heterogeneity. 95% CI was calculated, and due to the small number of included studies and low power of chi-square test for heterogeneity (in terms of the Cochrane handbook for systematic reviews of interventions), *p* < 0.1 was regarded as significant. Based on the results, sensitivity analysis and subgroup analysis will be performed if needed.

## Results

### General characteristics

**Fig. 1:**
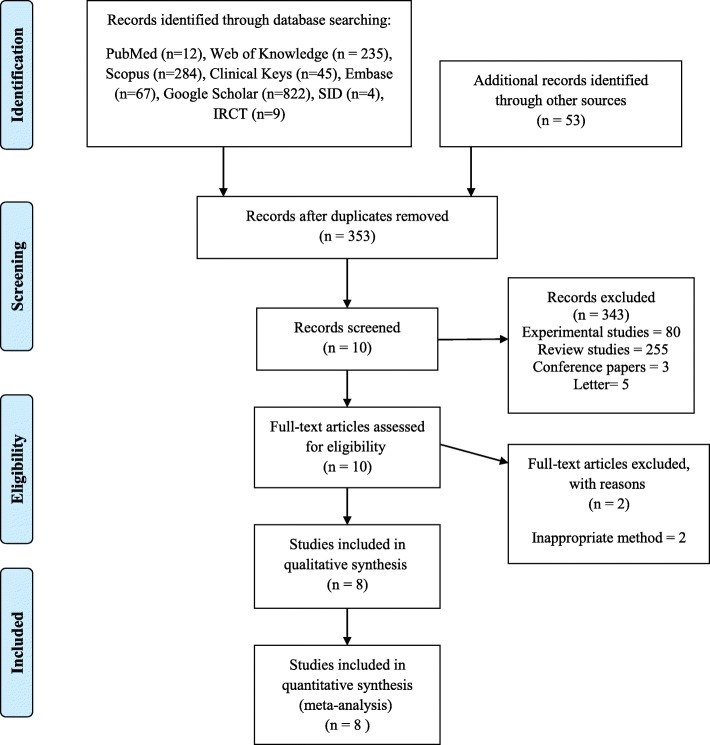
Flowchart of selection strategy and method (PRISMA statement)

We gathered 1531 studies from databases and other hand search sources. Eight of 1531 studies (Eight full-text papers under six trials) had eligibility criteria and were included in qualitative and quantitative analysis [33–40]. Three of them had the same ethic code (910732) (approval code of research ethics committee) and were different reports of one trial [33–35]. Also two other studies were similar and were different reports of one trial [36, 37]. We included all different outcomes (which considered in our systematic review) of the different reports of each trial to the meta-analysis (if the trial had multiple reports). The flowchart of searching and inclusion process of studies is shown in Fig. 1(According to PRISMA statement). Fore of included studies had used the date palm fruit [33, 36, 39, 40], and one study had used date fruit honey [38]. Descriptions and characteristics of the reviewed studies are shown in Table 1. No side effects were reported for date palm in any of the studies. The minimum and maximum intervention durations of founded studies were two days and four weeks, respectively. Date palm and its extracts were orally utilized in all of the studies. Sample sizes of these studies were 89 to 210 participants. 653 participants were included in the analysis, 325 participants in intervention group, and 328 in control group. Ages of participants were ranged from 20 to 40. All of these studies had control groups consisting of placebo and routine cares in one study [38], and routine cares in other studies [33, 36, 39, 40]. However, placebo control group was not included in meta-analysis and qualitative analysis.

**Table 1:** Characteristics of included studies (PICOS)

Number	Author/ Year	Study design	Participants and Sample size	Intervention	Control	Duration of intervention	Outcomes (considered in this study)	Results
1	Ahmed et al., 2018 [39]	Randomized, controlled, clinical trial	57 pregnant women 26 intervention group 31 control group	7 date’s fruits on the permission	Routine cares	Once	(1–3) Duration of first, second and third stage of labor	1. ↓ Duration of the first and the third stage of labor in intervention groups significantly compared with control group 2. ↑ Duration of the second stage of labor in intervention groups but not significantly compare with control group
2	Razali et al.,2017 [40]	Randomized controlled clinical trial	154 nulliparous singleton pregnant women 77 intervention group 77 control group	7 date’s fruits (approximately 80 g)	Routine cares	1–4 weeks	(1–3) Duration of first, second and third stage of labor (4) Frequency of caesarian section	1. ↓ Duration of the first and the third stage of labor in intervention groups but not significantly compare with control group 2. ↑ Duration of the second stage of labor in intervention groups but not significantly compare with control group 3. No significant difference between two groups in mode of delivery
3	Kariman and Jadidi et al., 2015 [36, 37]	Randomized, controlled, clinical trial	110 nuliparous pregnant women 55 intervention group 55 control group	7 date’s fruit per day	Routine cares	From week 38 of pregnancy to onset of delivery signs	(1) Duration of active phase of labor (2) Bishop score (3) Frequency of caesarian section	1. Significant ↓ of active phase of labor in intervention group compared with control group 2. Significant ↓in bishop score after intervention compared with control group 3. No significant differences in mode of delivery between two group 4. ↑ Bishop score significantly after intervention compared with control group 5. ↑ Cervical dilatation significantly after intervention compared with control group 6. ↑ Cervical effacement significantly after intervention compared with control group 7. ↑ Spontaneous labor significantly after intervention compared with control group
4	Kordi et al.,2013,2014, 2017 [33–35]	Quasi-randomized, controlled, clinical trial	210 pregnant women with gestational age of 37–38 weeks 105 intervention group 105 control group	70–75 g date’s fruit per day	Routine cares	1–3 weeks	(1) Bishop score (2) Frequency of caesarian section (3–5) Duration of first, second and third stage of labor	1. Significant ↑ of the mean Bishop Score in intervention group compared with control group 2. No significant difference between two groups in mode of delivery (↓ frequency of cesarean section in intervention group) 1. The average length of the second phase and the third phase in the intervention group was significantly lower than the control group 2. Spontaneous start of labor in the intervention group was significantly more than the control group 3. No significant difference between average length of active phase of labor in the two 4. ↑Cervical dilatation in intervention group compared with control group (significant) 5. ↓Length of pregnancy in intervention group compared with control group (significant)
5	Kordi et al.,2010 [38]	Randomized double blinded controlled clinical trial	60 nuliparous pregnant women with gestational age of 37–42 weeks 30 intervention group 30 control group	132 g date’s honey syrup from 4 cm cervix dilatation to labor	Routine cares	Maximum 1 day	(1) Duration of active phase of labor (2) Duration of second stage of labor	Significant ↓in duration of active and second phases of labor

### Risk of bias within studies (Figs. 2 and 3)

The details of risk of bias within included studies and authors judgment are listed in Table 2.

**Fig. 2:**
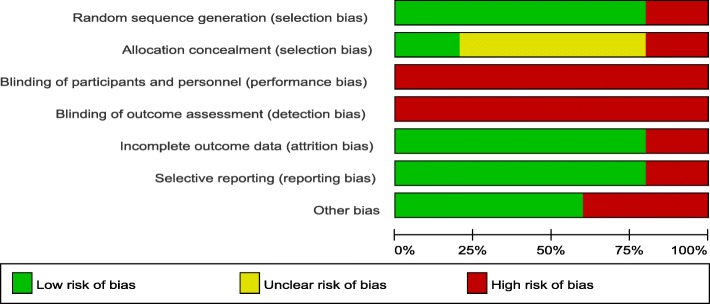
Risk of bias graph

**Fig. 3:**
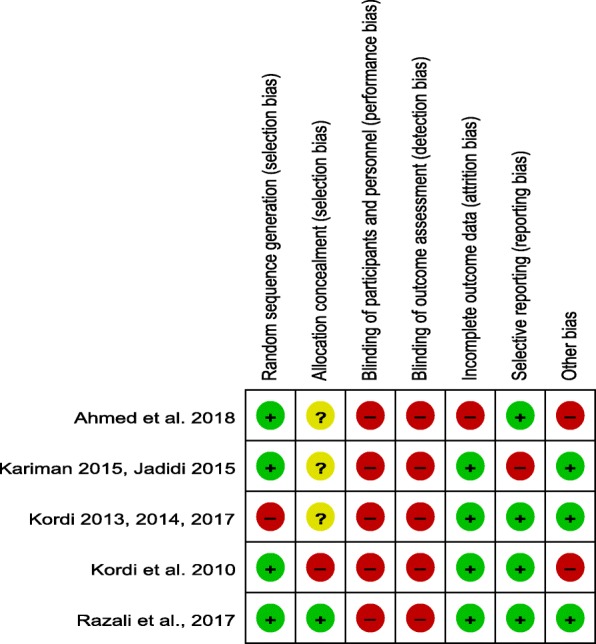
Risk of bias summary

**Table 2:** Risk of bias within studies

Bias	Authors judgment	Support for judgment
**Ahmed et al. (2018)** [39]		
Random sequence generation	Low risk	Simple random sampling has been used
Allocation concealment	Unclear risk	No specific information
Blinding of participants and personnel	High risk	Open label manner
Blinding of outcome assessors	High risk	Open label manner
Incomplete outcome data	High risk	Intention to treat analysis has not conducted.
Selective reporting	Low risk	Protocol is unavailable but the authors have reported their expected mentioned outcomes
Other	High risk	No registered protocol, sample size calculating method is not specified
**Razali et al. (2017)** [40]		
Random sequence generation	Low risk	Sealed envelope numbers has been used
Allocation concealment	Low risk	It was done using “sealed envelope” manner
Blinding of participants and personnel	High risk	Open label manner
Blinding of outcome assessors	High risk	Open label manner
Incomplete outcome data	Low risk	The dropped out has been mentioned and intention to treat has been analyzed
Selective reporting	Low risk	Protocol is unavailable but the both primary and secondary outcomes have been reported
Other	Low risk	Registered protocol exist, sample size calculating method is specified, Ethical approval exist, Specified inclusion and exclusion criteria, specified funding source, no conflict of interest
**Kariman and Jadidi et al. (2015)** [36, 37]		
Random sequence generation	Low risk	Random number generator has been used
Allocation concealment	Unclear risk	No specific information
Blinding of participants and personnel	High risk	Open label manner
Blinding of outcome assessors	High risk	Open label manner
Incomplete outcome data	Low risk	The dropped out has been mentioned and intention to treat has been analyzed
Selective reporting	High risk	Protocol is available but secondary outcomes have not been reported
Other	Low risk	Registered protocol exist, sample size calculating method is specified, Ethical approval exist, Specified inclusion and exclusion criteria, specified funding source, no conflict of interest
**Kordi et al.(2013, 2014, 2017)** [33–35]		
Random sequence generation	High risk	The days of the Week have been used for randomization
Allocation concealment	Unclear risk	No specific information
Blinding of participants and personnel	High risk	Open label manner
Blinding of outcome assessors	High risk	Open label manner
Incomplete outcome data	Low risk	The dropped out has been mentioned and intention to treat has been analyzed
Selective reporting	Low risk	Protocol is available and both primary and secondary outcomes have been reported
Other	Low risk	Registered protocol exist, sample size calculating method is specified, Ethical approval exist, Specified inclusion and exclusion criteria, specified funding source, no conflict of interest
**Kordi et al. (2010)** [38]		
Random sequence generation	Low risk	Simple random sampling has been used
Allocation concealment	High risk	There was no evidence for allocation concealment
Blinding of participants and personnel	High risk	Open label manner
Blinding of outcome assessors	High risk	Open label manner
Incomplete outcome data	Low risk	There was no lost to follow up
Selective reporting	Low risk	Protocol is unavailable but both primary and secondary outcomes have been reported
Other	High risk	Conflict of interest didn’t declared, no specified inclusion and exclusion criteria

#### Random sequence generation

Four studies of 5 studies had used random number table, random generator or computer programmed random sequencing; and thus, they were rated as low risk of bias. Another study used no reliable randomization method and was evaluated as high risk of bias.

#### Allocation concealment

One study of included studies had used sealed-envelopes method to the allocation concealment and was evaluated as low risk of bias. Three trials of five included trials did not determine the method of allocation concealment, and were evaluated as unclear risk of bias. One trial had not concealment and was evaluated as high risk of bias.

#### Blinding of participants and personnel

Due to the consumption of date fruit as intervention and routine care as control, all of the included studies had not performed blinding and were rated as high risk of bias.

#### Blinding of outcome assessment

All of these studies were assessed as high risk of bias; because they had no evidence of blinding of outcome assessors.

#### Incomplete outcome data

Out of 5 trials, 2 trials have mentioned the dropped out and analyzed the intention to treat, and 2 trials had no dropped out or lost to follow up; therefore they were rated as low risk of bias. Also, one study had attrition for missing participants; however, the statistical analysis was not followed by the intention to treat.

#### Selective reporting

One study had registered protocol; but the reported outcomes did not match with registered outcomes and were given the high risk of bias. Other included studies had reported their expected outcomes and were assessed as low risk of bias.

#### Other bias

Three trials had registered protocol, specified funding source, appropriate ethical criteria, inclusion and exclusion criteria, specified sample size calculating method, and declaration of conflict of interest; therefore, they were rated as low risk of bias. Other trials did not have some of mentioned cases and were rated as high risk of bias.

### Outcomes

We considered common outcome among the included studies in quantitative integration, included active phase of labor duration, first stage of labor duration, second stage of labor duration, and third stage of labor duration as primary outcomes; and bishop score and frequency of caesarian section as secondary outcomes. Also, we performed the sensitivity analysis, because one trial of included trials was quasi-randomized study. Sensitivity analysis did not change the results of primary meta-analysis. The summary of sensitivity analysis is shown in Table 3. The forest plots of sensitivity analyses are available in Appendix 2–6 (See Additional file 2). Moreover, we performed subgroup analysis for two subgroups (Intervention during labor, and intervention during pregnancy). The results of subgroup analysis showed significant changes in the third stage of labor meta-analysis results. The forest plots of subgroup analyses are available in Appendix 7–10 (See Additional file 3).

**Table 3:** The summary of sensitivity analyses

Number	Measured outcome	Meta-analyses of all studies (Overall effect statistical significance)	Sensitivity Analyses^a^ (Overall effect statistical significance)
1	Second stage of labor	P = 0.44	P = 0.64
2	Third stage of labor	P = 0.82	P = 0.39
3	Active phase of labor	**P = 0.01**^**b**^	**P = 0.01**^**b**^
4	Bishop score	**P < 0.00001**^**b**^	**P < 0.00001**^**b**^
5	Frequency of cesarean section	P = 0.23	P = 0.59

^a^quasi-randomized study (Kordi 2013, 2014, 2017) [33–35] removed from meta-analysis

^b^the significant values are shown with bold font

#### Active phase of labor

**Fig. 4:**
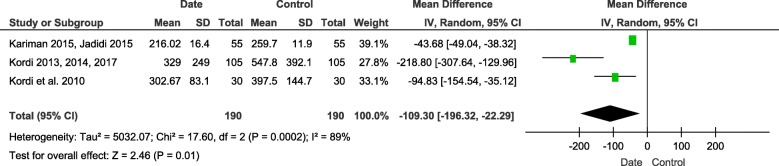
Forest plot of the duration of active phase of labor

Three trials with 380 participants were included (190 in intervention group and 190 in control group). There was moderate heterogeneity among the studies (I^2^ = 89%, *P* = 0.0002). The quantitative synthesis showed that date consumption significantly reduce the duration of active phase of labor compared with control group (MD = − 109.3, 95%CI (− 196.32, − 22.29), *P* = 0.01) (Fig. 4).

#### First stage of labor

**Fig. 5:**
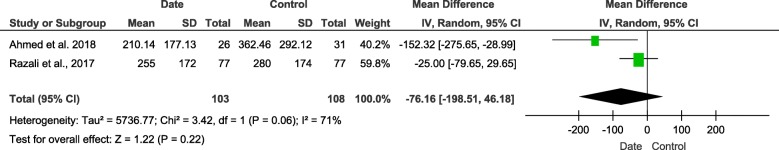
Forest plot of the duration of first stage of labor

Two studies reported the duration of first stage of labor. Totally, 211 participants were included (103 in intervention group and 108 in control group). Moderate heterogeneity accompanied (I^2^ = 71%, *P* = 0.06). There was no significant difference between two groups (MD = − 76.16, 95%CI (− 198.51, 46.18), *P* = 0.22) (Fig. 5).

#### Second stage of labor

**Fig. 6:**
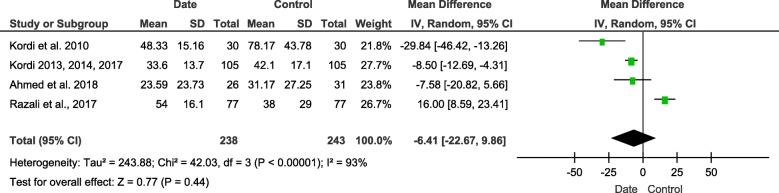
Forest plot of the duration of second stage of labor

We collected data from 4 trials with 481 participants (238 in intervention group and 243 in control group). The heterogeneity was high (I^2^ = 93%, *P* < 0.00001). There was no significant difference between two groups (MD = − 6.41, 95%CI [− 22.67, 9.86], *P* = 0.44) (Fig. 6).

#### Third stage of labor

**Fig. 7:**
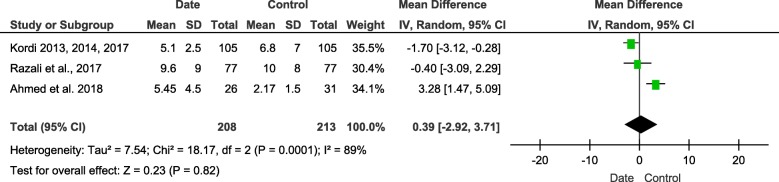
Forest plot of the duration of third stage of labor

Three trials with 421 participants (208 in intervention group and 213 in control group) described the duration of the third stage of labor. The intervention cannot decrease the duration of the third stage of labor (MD = 0.39, 95%CI (− 2.92, 3.71), *P* = 0.82). The heterogeneity was high (I^2^ = 89%, *P* = 0.0001) (Fig. 7). The subgroup analysis showed that the intervention during pregnancy significantly reduces the duration of the third stage of labor compared with control group. (Appendix 9) (See Additional file 3).

#### Bishop score

**Fig. 8:**
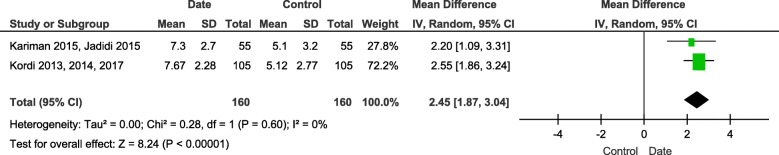
Forest plot of the bishop score

Two trials reported data on the bishop score with 320 participants (160 in intervention group and 160 in control group). There was no heterogeneity (I^2^ = 0%, *P* = 0.60). The intervention significantly improved the bishop score, compared to control group (MD = 2.45, 95%CI (1.87, 3.04), *P* < 0.00001) (Fig. 8).

#### Frequency of caesarian section

**Fig. 9:**
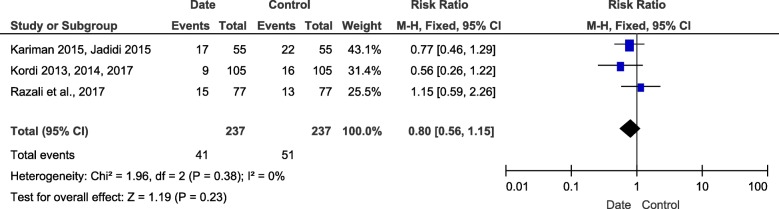
Forest plot of the caesarian section frequency

Three trials had showed the effect of intervention on frequency of caesarian section. 474 participants were included in this outcome meta-analysis (237 in intervention group and 237 in control group). Low heterogeneity was accompanied (I^2^ = 0%, *P* = 0.38). There was no significant difference between two groups. (Risk Ratio = 0.80, 95%CI (0.56, 1.15), *P* = 0.23) (Fig. 9).

#### Adverse effects

No side effects have been reported in any of the included studies.

## Discussion

Based on the performed searches, this is the first systematic review conducted on the effects of date on bishop score and frequency of caesarean section, and second one on the effects of date on the duration of labor stages. Meta-analysis showed that the consumption of date fruit can significantly reduce the duration of the active phase and improve the bishop score, and may reduce the frequency of cesarean section in intervention group compared to control group. Based on meta-analysis, date palm fruit consumption resulted in a significant reduction in the duration of active phase of labor compared with control group; however, it is not observed in the first, second, and the third stage of labor.

Nasiri et al. in their systematic review have achieved different results about the duration of labor stages. Those results are not reliable, because they had included three duplicated published studies in their meta-analysis. In that study, bishop score and frequency of caesarean section were not investigated [32].

Different mechanisms have been expressed on the effects of date palm on the labor process. Date palm fruit has high calorie, and is proposed as an energizer. The sugar in date fruit is glucose with simple digestion and absorption. Date fruit provides and maintains required energy for pregnant woman to prevent tiredness. It leads to normal progress of labor, due to providing continual glucose and preserving body electrolytes [23, 25]. Oxytocin and prostaglandins have been widely utilized for ripening of cervix, stimulation of uterine contractions, and induction and stimulation of labor especially when the duration of latent phase of labor has been expanded [41, 42]. Misuse of oxytocin and prostaglandins and insufficient maternal care in labor, can lead to delivery complications [43, 44]. Myometer oxytocin receptors are increasing in the last weeks of pregnancy. Estrogen and progesterone levels change in the 34–35 weeks of pregnancy that these changes could lead to the improvement of irritability of uterine, improvement of responsiveness of uterine to contractor factors, and the improvement of cervical preparation to labor [45]. Therefore, date fruit consumption in last weeks of pregnancy can cause labor induction and stimulation, because date fruit acts on prostaglandin receptors, causes early stimulation of uterine contractions, and improves response to syntocinon if it is necessary [41]. Fatty acids in date palm in addition to production and reservation of energy play important role in the prostaglandins production, and following that, in the reinforcement of uterine muscles [26, 27, 46]. On the other hand, drinking water after consuming date fruit during labor is effective on labor progress and shortens the second and the third stages of labor [47]. As well as date palm can increase antioxidant capacity for 4 h, and following that increases pain tolerance, this results in reduction of the first and the third stages of labor [22, 26]. Also, date fruit has anti-inflammatory and antioxidant properties and it is rich in calcium, serotonin, and tannin, and can play a role in contraction of smooth muscles of uterine [22, 48]. As mentioned earlier, consumption of date fruit had significantly increased the bishop score and cervical dilatation. Bishop score (that contains cervical dilatation and …) is known as evaluation criteria of labor progress and the increasing factor of normal vaginal labor [49, 50]. Lack of preparation of cervix and induction of labor can lead to increase of caesarean section rate and postpartum hemorrhage [51–53]. However, increase of the bishop score and preparation of cervix, can increase normal vaginal labor rate and reduce the caesarean section rate [54]. The low dose of oxytocin causes the improvement of the bishop score and the preparation of cervix [55], thus the consumption of date fruit affects the improvement of the bishop score by increasing the activity of myometer contraction. This is a confirmation of possible hypothesis of oxytocin in date fruit and its effect on uterine muscle contraction [22, 24, 56]. Also, date fruit contains saturated and unsaturated fatty acids linoleic, oleic, and stearic. Linoleic acid breaks down to the arachidonate, and then to the eicosanoid. The eicosanoids finally convert to the prostaglandins and they improve the preparation of cervix by increasing subserosal fluid and making changes in collagen bands, and they cause the increasing of sensitivity of uterine to oxytocin [36, 48, 57].

Generally, the mechanism of cervical preparation is unknown; however, changes in levels of estrogen and progesterone, increasing of the prostaglandins production, increasing of myometer sensitivity to oxytocin and prostaglandins, and their interactions are identified to be effective [45, 54].

Up to the best of our knowledge, the most common causes of cesarean section are abnormal progress of labor and non-effective contractions of uterine [11, 12, 16]. Lack of eating energizer foods during labor lead to the increasing of non-effective contractions of uterine and following that increasing of the augmented delivery and increasing the cesarean section rate [20]. Limited reserves of glycogen and body fluids in pregnant women that have limited consumption of food and liquids during labor can cause low perfusion and low nutrition of uterine, which can be followed by abnormal labor progress, prolonged labor, and the increasing rate of cesarean section [36].

### Limitations

One of the limitations of this study was the lack of specified standard for the kind and the amount of date fruit that must be consumed to bring positive effects on labor progression and cervix preparation. Also, it was not determined that, how long at what intervals of consumption of date fruit is needed to energy supply during labor, reduce the cesarean section rate and promotion of labor outcomes.

Another limitation of this study was the high risk of bias in performed studies. Especially, due to the methods of studies and using the date palm fruit in the studies, blinding of participants and researchers was not possible. Therefore, for this reason, performance bias was raised in included studies. Furthermore, none of these studies had outcome assessor blinding.

The other limitation of this study was the unknown time of intervention and duration of intervention in the most studies.

## Conclusion

Despite widespread utilizing of date palm, there is not enough clinical evidence to support the clinical effects, which were mentioned in review articles and traditional medical systems. Based on this study result, date fruit can reduce the duration of active phase and improve the bishop score. The growing trend of recent studies about date palm provides scientific rationale for date palm clinical abilities; however, due to from the low to mediate quality of the studies, it seems that the other studies are needed to prove these results better than this.

## Supplementary information

**Additional file 1.** Appendix 1: Sample search strategy used for PubMed.

**Additional file 2.** Appendices 2–6: Forest plots of sensitivity analysis.

**Additional file 3.** Appendices 8–10: Forest plots of subgroup analysis

## Data Availability

All data generated during this study are included in this article, tables, figures and its supplementary information files. Link/references of databases used in this study: www.clinicalkey.com, www.embase.com, www.scopus.com, www.ncbi.nlm.nih.gov/pubmed, www.webofknowledge.com, www.sid.ir, www.irct.ir, www.scholar.google.com The public access of PubMed, Google Scholar, Scientific Information Database (SID), and IRCT (Iranian Registry of Clinical Trials) are open but Scopus, Web of Knowledge, Clinical Keys, and Embase are close. We used institutional access of Tabriz University of Medical Sciences for these databases.

## Abbreviations

WHO: World health organization
IVF: in vitro fertilization
IUI: Intrauterine insemination
Kcal: Kilo calories
PRISMA: Preferred reporting items for systematic reviews and meta-analyses
SID: Scientific Information Database
IRCT: Iranian Registry of Clinical Trials
EAGLE: European Association for Grey Literature Exploitation
HMIC: Health Care Management Information Consortium
RR: Risk ratio
MD: Mean difference
