# Supplementary material for: Effects of date fruit (Phoenix dactylifera L.) on labor and delivery outcomes: a systematic review and meta-analysis
Source: BMC Pregnancy Childbirth. 2020 Apr 14;20:210. doi: 10.1186/s12884-020-02915-x (PMC7157989; doi:10.1186/s12884-020-02915-x)
Supplement: Supplementary file 1 — Additional file 1. Appendix 1: Sample search strategy used for PubMed. [file 12884_2020_2915_MOESM1_ESM.docx]

| Database: Pubmed  Date of last search: August 2019 |
| --- |
| Search strategy:  (((((date[Title/Abstract] AND palm)[Title/Abstract] OR ( phoenix[Title/Abstract] AND dactylifera)[Title/Abstract] OR (date[Title/Abstract] AND fruit)[Title/Abstract])) AND ((labor)[Title/Abstract] OR (delivery)[Title/Abstract])) AND Clinical Trial[ptyp]) |
| Number of results: 12 |

Appendix 1: Electronic search strategy
