# Supplementary material for: Effects of date fruit (Phoenix dactylifera L.) on labor and delivery outcomes: a systematic review and meta-analysis
Source: BMC Pregnancy Childbirth. 2020 Apr 14;20:210. doi: 10.1186/s12884-020-02915-x (PMC7157989; doi:10.1186/s12884-020-02915-x)
Supplement: Supplementary file 2 — Additional file 2. Appendices 2–6: Forest plots of sensitivity analysis. [file 12884_2020_2915_MOESM2_ESM.docx]

**Sensitivity Analysis**

Appendix 2: Forest plot of the duration of second stage of labor (sensitivity analysis)

Appendix 3: Forest plot of the duration of third stage of labor (sensitivity analysis)

Appendix 4: Forest plot of the duration of active phase of labor (sensitivity analysis)

Appendix 5: Forest plot of the frequency of cesarean section (sensitivity analysis)

Appendix 6: Forest plot of the bishop score (sensitivity analysis)
