# Supplementary material for: Effects of date fruit (Phoenix dactylifera L.) on labor and delivery outcomes: a systematic review and meta-analysis
Source: BMC Pregnancy Childbirth. 2020 Apr 14;20:210. doi: 10.1186/s12884-020-02915-x (PMC7157989; doi:10.1186/s12884-020-02915-x)
Supplement: Supplementary file 3 — Additional file 3. Appendices 8–10: Forest plots of subgroup analysis [file 12884_2020_2915_MOESM3_ESM.docx]

**Subgroup analysis**

Appendix 7: Forest plot of the duration of first stage of labor (subgroup analysis)

Appendix 8: Forest plot of the duration of second stage of labor (subgroup analysis)

Appendix 9: Forest plot of the duration of third stage of labor (subgroup analysis)

Appendix 10: Forest plot of the duration of active phase of labor (subgroup analysis)
